# Ectopic CXCR2 expression cells improve the anti-tumor efficiency of CAR-T cells and remodel the immune microenvironment of pancreatic ductal adenocarcinoma

**DOI:** 10.1007/s00262-024-03648-y

**Published:** 2024-03-02

**Authors:** Zhengjie Dai, Xuan Lin, Xu Wang, Xuan Zou, Yu Yan, Ruijie Wang, Yusheng Chen, Yesiboli Tasiheng, Mingjian Ma, Xu Wang, He Cheng, Xianjun Yu, Chen Liu

**Affiliations:** 1 Department of Pancreatic Surgery, Fudan University Shanghai Cancer Center, No. 270 Dong An Road, Xu-Hui District, Shanghai, 200032 China; 2 Department of Oncology, Shanghai Medical College, Fudan University, Shanghai, 200032 China; 3 Shanghai Pancreatic Cancer Institute, Shanghai, 200032 China; 4 Pancreatic Cancer Institute, Fudan University, Shanghai, 200032 China; 5 Cancer Research Institute, Shanghai Key Laboratory of Radiation Oncology, , Fudan University Shanghai Cancer Center, Fudan University, Shanghai, China

**Keywords:** CXCR2, Cell therapy, Pancreatic ductal adenocarcinoma, Tumor microenvironment

## Abstract

**Background:**

Recent progressions in CAR-T cell therapy against pancreatic ductal adenocarcinoma (PDAC) remain disappointing, which are partially attributed to the immunosuppressive microenvironment including macrophage-mediated T cell repletion.

**Methods:**

We first characterized the expression patterns of macrophage-relevant chemokines and identified CXCR2 as the key factor regulating T cell trafficking and tumor-specific accumulation in PDAC microenvironment. After that, we synthesized and introduced a CXCR2 expression cascade into Claudin18.2 CAR-T cells and compared the behaviors of CAR-T cells in vitro and in vivo. The therapeutic potential of CXCR2 CAR-T was evaluated in two different allogeneic models: subcutaneous allografts and metastatic PDAC models.

**Results:**

The results showed that CXCR2 CAR-T not only reduced the size of allografted PDAC tumors, but also completely eliminated the formation of metastases. Lastly, we investigated the tumor tissues and found that expression of ectopic CXCR2 significantly improved tumor-targeted infiltration and residence of T cells and reduced the presence of MDSCs and CXCR2 + macrophages in PDAC microenvironment.

**Conclusion:**

Our studies suggested that ectopic CXCR2 played a significant and promising role in improving the efficiency of CAR-T therapy against primary and metastatic PDAC and partially reversed the immune-suppressive microenvironment.

**Supplementary Information:**

The online version contains supplementary material available at 10.1007/s00262-024-03648-y.

## Introduction

Pancreatic ductal adenocarcinoma (PDAC) is one of the most devastating malignancies characterized by a grim prognosis, primarily attributable to systemic metastasis and high recurrence rates [1, 2]. For the patients with advanced and metastatic pancreatic cancer, conventional treatments such as surgery and chemotherapy often provide limited benefits, but some innovative treatments, including cellular therapies, may present new hope [3–5]. Chimeric Antigen Receptor T-cells (CAR-T) therapy has demonstrated significant clinical efficacy in the treatment of a range of hematological malignancies, but their therapeutic potential in the realm of solid tumors remains constrained [6, 7]. The employment of CAR-T cells for the treatment of solid tumors presents several specific challenges, including a lack of suitable tumor targets, the immunosuppressive nature of the tumor microenvironment (TME), tumor heterogeneity and the limited migratory and persistence capabilities of CAR-T cells within solid tumors [8, 9].

Recently, the treatment of PDAC with CAR-T cells targeting CLDN18.2 has thus far displayed marginal benefits [10–12]. The insufficient T cell infiltration and the abundance of immune-suppressive cells in TME were responsible for the disappointing outcome [13]. On the one hand, T cell infiltration is regulated by self-receptor compatibility with chemokine networks (CXCLs, CCLs, etc.). On the other hand, the immune-suppressive immune cells, represented by MDSCs and macrophages, also participate in the secreting and responding of cytokines, and are thus actively involved in regulating T cell infiltration [14, 15].

Interestingly, macrophages and T cells displayed distinct infiltration patterns in PDAC microenvironment. It can be inferred that differences in chemokine compatibility between macrophages and T cells are responsible for their infiltration patterns, and it is unknown whether the transfer of certain chemokine receptors of macrophages into T cells can improve T cell trafficking. CXCR2 is a compelling chemokine receptor, and its ligands CXCL5 and CXCL8 are primarily expressed in PDAC [16, 17]. CXCR2 is closely associated with the response and recruitment of immune cells. Besides, CXCR2 is not expressed in circulating T cells, yet its presence has been linked to macrophage infiltration [18]. Previous research also suggested that introduction of certain CXCRs may significantly improve the therapeutic effectiveness of CAR-T therapy in several different types of cancer [19–22]. Nevertheless, the potential beneficial functions of CXCR2 expression in CAR-T cells for the treatment of PDAC demand further investigation.

Here, we first confirmed the CXCL5-CXCR2 axis as one of the effectors responsible for macrophage-specific migration into pancreatic cancer. With a hope of transferring the migration pattern of macrophages into CAR-T cells, we generated lentivirus encoding an anti-CLDN18.2 CAR and a mouse version of CXCR2 (CXCR2 CAR) and evaluated the anti-tumor potential of the CAR-T cells both in vitro and in vivo. Furthermore, a primary organoid model was also employed to validate the in vitro killing functions of CAR-T cells. The recruitment of CAR-T cells at the tumor site was significantly enhanced with the aid of ectopic CXCR2, and CXCR2 CAR-T cells also demonstrated superior effectiveness in treating PDAC compared to control counterparts, with an increased proportion of effective memory T cells. Furthermore, CXCR2 CAR-T cell therapy reduced the recruitment of MDSCs and the presence of CXCR2-positive macrophages, partially reversing the immunosuppressive microenvironment. Together, the ectopic CXCR2 expression promoted the survival of CAR-T cells and enhanced their anti-tumor efficacy. Additionally, CXCR2 CAR-T significantly inhibited metastases of PDAC cells into livers and improved survival. Together, the aforementioned studies suggested that ectopic CXCR2 in CAR-T cell therapy played a significant and promising role in treating primary and metastatic PDAC and partially reversed the immune-suppressive microenvironment.

## Results

### A large amount of CXCL5 secreted by PDAC in the PDAC tissue recruits the migration of CXCR2 + macrophages

**Fig. 1:**
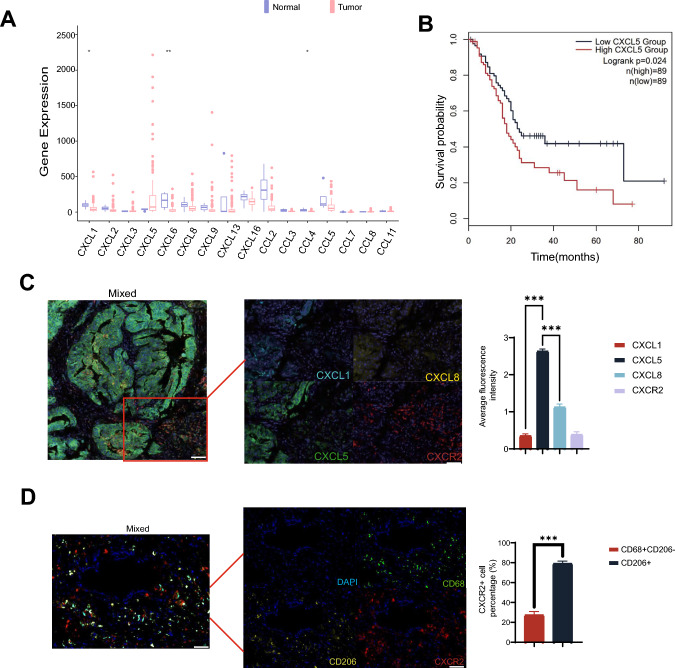
Expression of CXCL5 and distribution of CXCR2 + macrophage in PDAC TME. **A** Chemokine expression in pancreatic tumors and normal tissues was evaluated using data derived from the TCGA database. **B** Survival of PDAC patients with high and low CXCL5 expression. **C** Representative images from multiplex fluorescence immunohistochemistry highlight the spatial distribution of CXCL1, CXCL5, CXCL8, and CXCR2 in pancreatic cancer tissue samples. The distribution was analyzed using multiplex fluorescence immunohistochemistry, and fluorescence intensities were determined.Scale bars, 400 μm in Mixed. Scale bars, 100 μm in other figure. **D** Representative images from multiplex fluorescence immunohistochemistry depict the spatial distribution of CD68, CD206, and CXCR2 in tissue samples from pancreatic cancer patients. The distribution was assessed using multiplex fluorescence immunohistochemistry, and the count of CXCR2 + CD68 + cells was obtained relative to CD206 + cells. Scale bars, 100 μm. Data are expressed as mean ± SEM from triplicate experiments. Statistical significance is indicated as follows: *P* < 0.05, *P* < 0.01, **P < 0.001

Among all CXCL and CCL family members, only CXCL5 was significantly upregulated in PDAC compared with normal tissues, and only CXCL6 was significantly downregulated in PDAC. Furthermore, survival analysis on TCGA cohort indicated that CXCL5 is positively correlated with OS (Fig. 1A). A subsequent screening for chemokines whose expression levels in the pancreas correlated with survival differences identified CXCL5 as a potential therapeutic target (Fig. 1B). We further validated the specific elevation of CXCL5 through immunohistochemistry and multiplex immunohistochemistry in our patient cohort. Notably, CXCR2, the receptor for CXCL5, was predominantly expressed not in PDAC cells, but in mesenchymal stromal cells (Supplementary Fig. S1A, C). To investigate the potential migratory association of CXCR2 with immune cells within the PDAC immune microenvironment, we further examined the expression of CXCR2 on CD68-positive cells (total macrophage population) and CD206-positive cells (M2 macrophages) using multiplexed immunohistochemistry. Our results indicate that CXCR2 is widely expressed on the total macrophage population with M2 macrophages (Fig. 1D). Additionally, to examine and validate the correlation between CXCR2 and macrophage migration in PDAC, we explored the immune infiltration correlation of CXCR2 and its corresponding chemokines using the TIMER 2.0 database. This analysis revealed a positive correlation between CXCR2 expression and the infiltration of macrophages and CD8 + T cells (Supplementary Fig. S1B). Concurrently, we assessed the extent of influence that chemotactic factors and the CXCR2 inhibitor exert on the chemotactic abilities of macrophages in PDAC (Supplementary Fig. S1C). Taken together, these results suggest that PDAC may employ an immune cell infiltration strategy involving the secretion of CXCL5 to recruit CXCR2 + macrophage subpopulations. This strategy holds potential for boosting the infiltration capacity of CAR-T cells.

### Generation of CLDN18.2-specific CAR-T cells co-expressing CXCR2

**Fig. 2:**
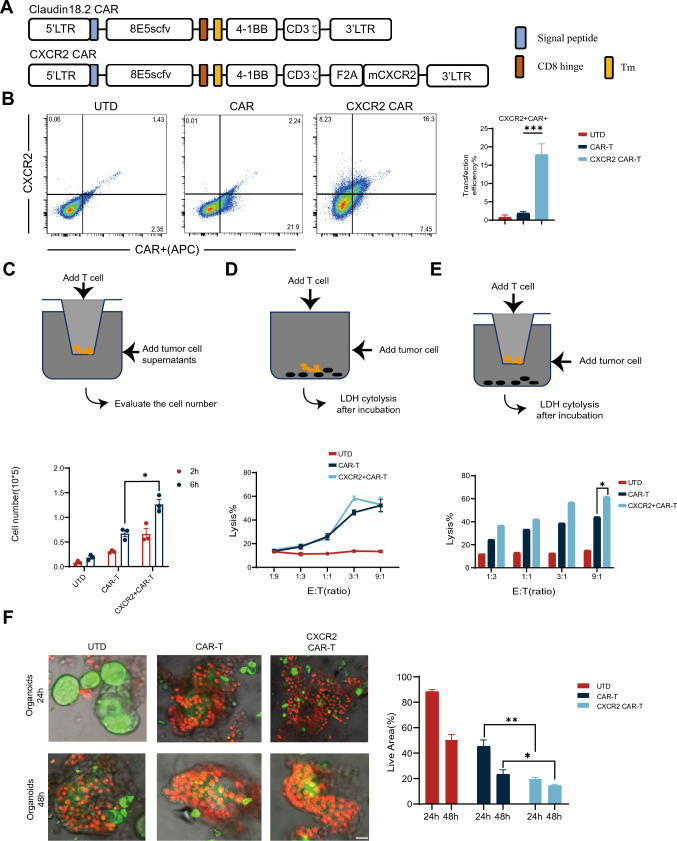
Generation of CLDN18.2-specific CAR-T cells co-expressing CXCR2. **A** This panel provides a schematic representation of components of the Claudin18.2-targeted conventional and CXCR2 CARs. The construct includes an extracellular antigen recognition domain, a transmembrane domain (TM), an intracellular segment of mouse 4-1BB costimulatory molecules, and a mouse CD3-ζ chain. **B** Transduction efficiency and CXCR2 expression in both CAR and CXCR2 CAR were determined by Fluorescence-Activated Cell Sorting (FACS) using T cells sourced from C57BL/6 mice. UTD served as negative controls. **C** CAR-T cells and CXCR2 CAR-T cells were introduced into the upper compartment of a Transwell plate. KPC cell culture supernatant, filtered to remove cell debris, was added to the lower compartment of the plate. The number of cells that migrated to the lower chamber was quantified either 2 or 6 h later. **D** CAR-T cells were co-incubated with KPC cells at varying effector-to-target (E:T) ratios for a duration of 18 h. Cell lysis was determined using standard non-radioactive cytotoxicity assays. **E** CAR-T cells and KPC cells were incubated together at varying E:T ratios for 18 h in both the upper and lower regions of a Transwell plate. A matrix gel was used to simulate the infiltration process across a filter mesh membrane. Cell lysis was evaluated using a standard non-radioactive cytotoxicity assay. **F** Representative fluorescent staining images of organoid effects by CAR-T. CAR-T cells were co-cultured with pancreatic cancer patient-derived organoid models. The cytotoxic effects of CAR-T cells were evaluated using confocal microscopy and AO/PI staining at 24 and 48 h time points. Scale bars, 50 μm. Data are expressed as mean ± SEM from triplicate experiments. Statistical significance is indicated as follows: *P* < 0.05, *P* < 0.01, **P < 0.001

A mouse version of 2nd generation CAR cascade was synthesized by fusing CLDN18.2-specific scFv with mouse 4-1BB and CD3-ζ intracellular signaling structural domains. Additionally, we developed another construct carrying the CAR cascade and mouse CXCR2 linked via an F2A peptide sequence (CXCR2 CAR) (Fig. 2A). CAR-T cells were transduced at 23.7% efficiency and CXCR2 cells at 24.5% efficiency (Fig. 2B), which is determined by flow cytometry. Subsequently, we evaluated the migratory capability of CXCR2 using the transwell migration experiment. It was observed that a greater number of CXCR2 CAR-T cells migrated to the lower chamber compared to both CAR-T cells and untransduced T cells (UTD) (Fig. 2C). Then, we screened the Claudin18.2 positive KPC cell line and PANC02 cells and evaluated the cytotoxicity of CAR-T cells under conditions with and without migration resistance. The cytotoxicity experiments showed no significant difference in the killing ability of CXCR2 CAR-T cells and CAR-T cells in the absence of migration resistance. However, with the introduction of migration resistance, the cytotoxic capability of CXCR2 CAR-T cells was significantly superior to that of CAR-T cells (Fig. 2D, E).To further assess the cytotoxic effectiveness of CXCR2 CAR-T cells against tumor cells, we developed a humanized organoid model and substituted mouse CXCR2 with human CXCR2. The cytotoxic efficiency was evaluated against organoids at various time points using AO/PI staining. The results demonstrated that CXCR2 CAR-T cells had a higher killing efficiency compared to CAR-T cells across all tested time points (Fig. 2F).

### CXCR2 enhanced anti-tumor effect of CAR-T cells in PDAC allografts

**Fig. 3:**
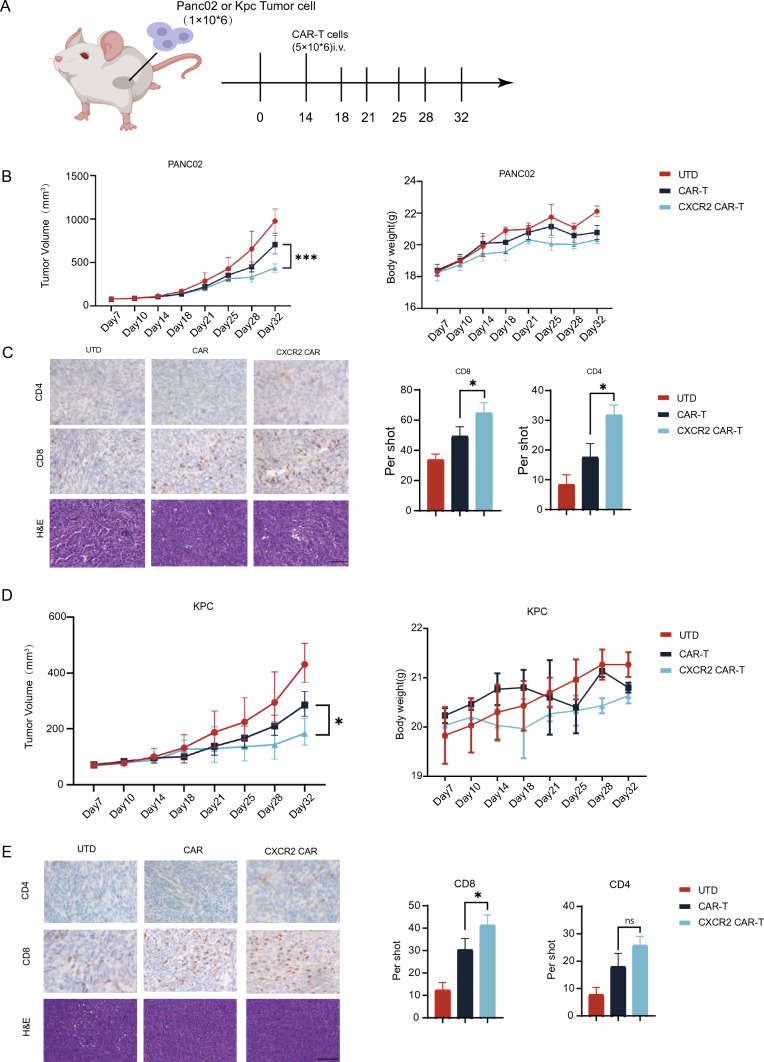
CXCR2 enhanced anti-tumor effect of CAR-T cells in pancreatic cancer. **A** An in vivo anti-tumor assay was conducted, wherein C57BL/6 mice were inoculated with either PANC02 or KPC cells and subsequently administered with CAR-T cells intravenously (at least five mice per group). **B** Tumor volume and body weight were monitored in the PANC02 group. **C** Tumor tissues from the PANC02 group were analyzed for the infiltration of CD4 and CD8 cells following CAR-T cell treatment. Random fields were selected for cell counting under the same area. Scale bars, 100 μm. **D** Tumor volume and body weight were assessed in the KPC group. **E** In the KPC group, tumor tissues were analyzed for the infiltration of CD4 and CD8 cells post-CAR-T cell treatment, and cell counting was conducted in random fields under the same area. Scale bars, 100 μm. Data are expressed as mean ± SEM from triplicate experiments. Statistical significance is indicated as follows: *P* < 0.05, *P* < 0.01, **P < 0.001

**Fig. 4:**
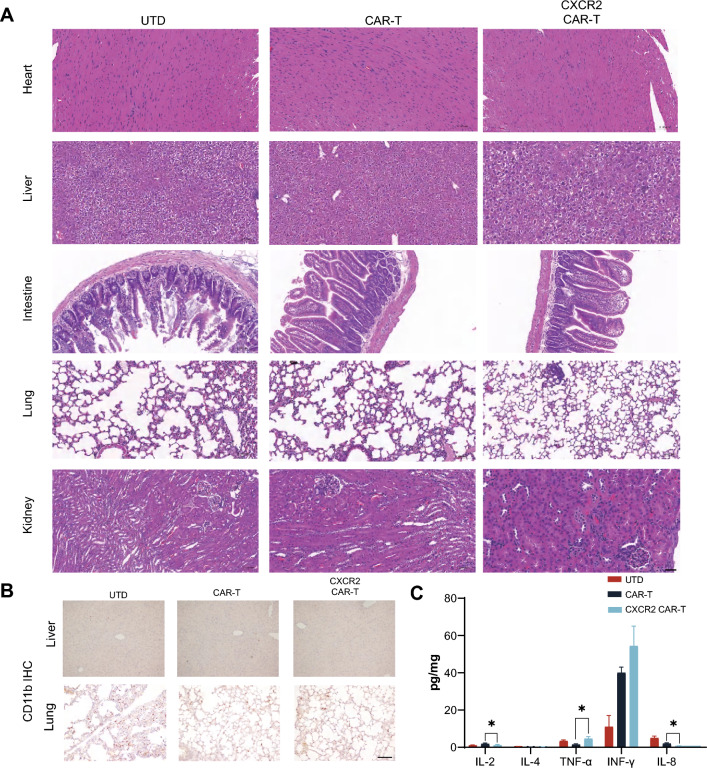
Evaluation of side effects and toxicity of CXCR2 CAR-T cells. **A** H&E staining results of heart, liver, intestinal, lung and kidney tissue sections of each experimental group. **B** Representative immunostaining images of liver CD11b + cells in each treatment group. **C** Secretion levels of IL-2, IL-4, IL-8, TNF-*α*, and IFN-γ in each experimental group. All data are mean ± SEM of triplicate experiments. Scale bars, 200 μm. Data are expressed as mean ± SEM from triplicate experiments. Statistical significance is indicated as follows: *P* < 0.05, *P* < 0.01, **P < 0.001

To examine the anti-PDAC activity of CXCR2 CAR-T, we developed mouse models with Claudin18.2-positive tumor syngeneic grafts derived from two distinct cell lines (Fig. 3A). On day 14, we compared tumor volumes and, finding no significant differences, grouped the mice for treatment with UTD, CAR-T, and CXCR2 CAR-T. The results revealed that in both models, regardless of cellular origin, the group treated with CXCR2 CAR-T demonstrated a statistically significant deceleration of tumor growth from day 28 compared to the CAR-T group (Fig. 3B, D). There was no notable difference in the body weight of mice across all groups. Furthermore, we collected tumor samples for immunohistochemistry analyses. We observed the increased infiltration of CD8 + T cells in all CXCR2 CAR-T groups comparing with CAR-T groups. In the PANC02 cell-derived tumor model, there was an increase in infiltration of CD4 + T cells in the CXCR2 CAR-T group. However, no statistically significant difference in infiltration degree was detected in the KPC cell-derived tumor model (Fig. 3C, E). Concurrently, H&E staining of tissue sections from the heart, liver, intestines, lung, and kidneys did not reveal any morphological abnormalities after CAR-T cell injection (Fig. 4A). Additionally, CD11b staining in other organs did not exhibit an increase in the quantity of CD11b + cells, which suggest that CAR-T cell therapy did not precipitate severe toxicity (Fig. 4B). Then, we measured the expression of various cytokines in tumor tissues by ELISA and found that there was a statistical difference in IL-8 along with a group-by-group decreasing trend in UTD group, CAR-T group, and CXCR2 CAR-T group (Fig. 4C). These findings suggest CXCR2 expression enhances the anti-tumor efficacy of CAR-T cells by promoting T cell infiltration into tumors.

### CXCR2 CAR-T cell therapy diminishes the mobilization of MDSCs and CXCR2 + macrophages in the tumor tissue of mice afflicted with PDAC

**Fig. 5:**
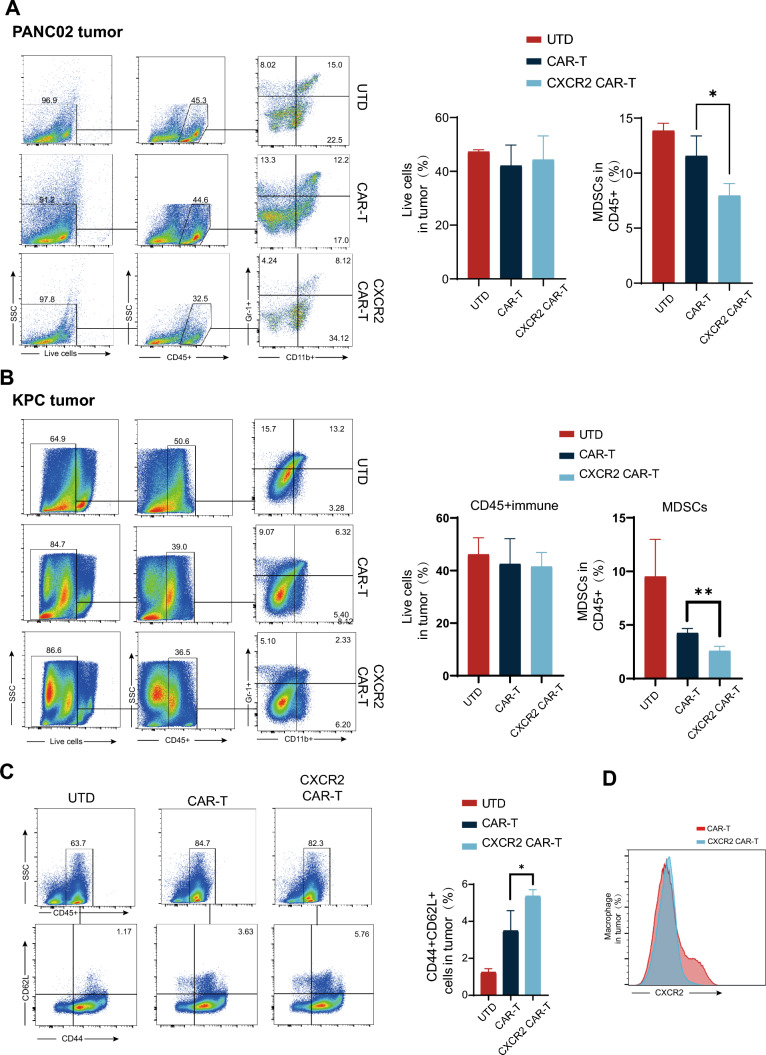
CXCR2 CAR-T Cell Therapy decreased the number of MDSCs and CXCR2 + Macrophages in PDAC. **A** Representative flow cytometry graphs illustrated the frequency and count of CD45 + immune cells and MDSCs in tumor tissues across various treatment groups in PANC02-inoculated mice. **B** Representative flow cytometry graphs displayed the frequency and number of CD45 + immune cells and MDSCs in tumor tissues across various treatment groups in PANC02-inoculated mice. **C** Representative flow cytometry graphs illustrating the proportion of CD44 + cells versus CD62L + cells among splenocytes. **D** Representative flow cytometry graphs exhibiting the ratio of CD44 + to CD62L + cells in the CXCR2 CAR-T treatment group, along with the expression of CXCR2 in macrophage cells located within tumor tissues in the CAR-T treatment group. Data are expressed as mean ± SEM from triplicate experiments. Statistical significance is indicated as follows: *P* < 0.05, *P* < *0.01*, **P < 0.001

In order to evaluate the impact of CXCR2 CAR-T cell therapy on the variation of immune cell components within the PDAC microenvironment, we quantified the number of MDSCs in murine tumors via flow cytometric analysis. The results indicated a decline in the number of infiltrated MDSCs in both tumor models from two different cell origins in the CXCR2 CAR-T group compared to the CAR-T group (Fig. 5A, B). Further, we isolated spleen cells from mice and quantified the number of CD44 + CD62L + cells through flow cytometry, which revealed the formation of cellular memory in both CAR-T and CXCR2 CAR-T groups (Fig. 5C). Simultaneously, we observed that the ratio of CD4 + to CD8 + T cells in the circulatory system remained unaltered (Supplementary Fig. S1B). We also determined the proportion of CXCR2 + cells in the macrophage subset. Interestingly, we observed a decrease in the infiltration of CXCR2 + macrophages in the CXCR2 CAR-T group, suggesting a specific alteration of CXCR2 + macrophage infiltration by the CXCR2 CAR-T therapy (Fig. 5D).

### CXCR2 CAR-T cell therapy inhibits the growth and formation of PDAC metastases

**Fig. 6:**
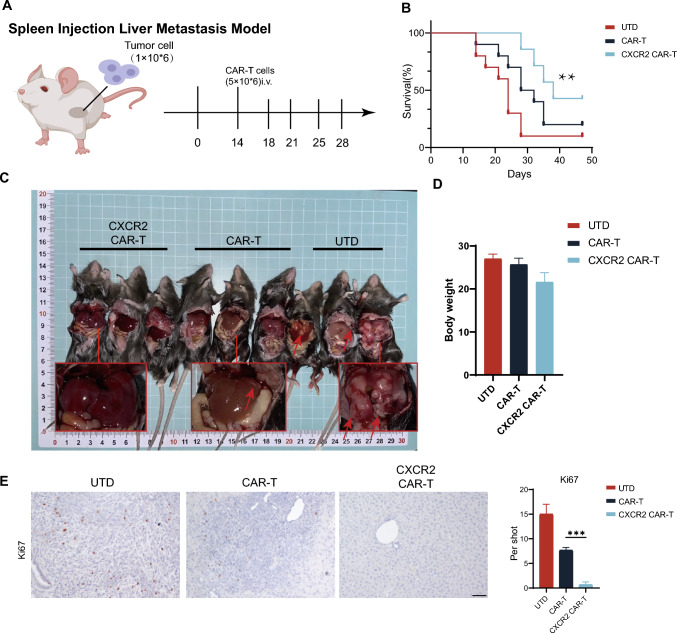
CXCR2 CAR-T cell therapy inhibited the growth and formation of PDAC metastases. **A** A pancreatic cancer liver metastasis model was established via splenic injection, and CAR-T treatment was administered on day 14. **B** The survival timeline for mice across different treatment groups is presented. **C** On day 28, dissections revealed liver metastases in the mice, and comparisons of mouse weights across various groups were made. **D** Representative images depict Ki67 expression in liver tissues from the mice, and counts of Ki67 + cells were made according to distinct tissue distributions. Scale bars, 200 μm. Data are expressed as mean ± SEM from triplicate experiments. Statistical significance is indicated as follows: *P* < 0.05, *P* < 0.01, **P < 0.001

Our findings indicate a variation in the expression of CXCL5 and CXCR2 between primary foci and metastases in pancreatic cancer (Supplementary Fig. S1D). To assess the therapeutic potential of CXCR2 CAR-T cell therapy for PDAC metastases, we established a hepatic metastasis model of PDAC through splenic injection (Fig. 6A). Initially, we examined variations in murine survival in PDAC metastases and discovered that the CXCR2 CAR-T group significantly extended the survival of mice. Although the CAR-T group also improved survival time relative to the Untreated (UTD) group, the observed increase in tumor load and bleeding resulting from tumor invasion into the abdominal arteries after 40 days suggested an insufficient suppression of metastatic tumorigenesis in the CAR-T group (Fig. 6B). As mice within the UTD group demonstrated noticeable tumor-induced mortality starting from day 18, we opted to euthanize and dissect a select number of mice on day 28 to further examine metastatic tumor progression and subsequently harvested their livers for immunohistochemical analysis (Fig. 6C). Concurrently, we monitored the mice's body weights; the marginal increase in the body weights of mice in the UTD group when compared to the CAR-T group could be attributed to the tumor load (Fig. 6D). We utilized Ki-67 as an indicator of proliferating metastatic tumor cells and counted Ki-67 positive cells in the immunohistochemical assays. Our findings indicated that the CXCR2 CAR-T group significantly curtailed the advancement of liver metastases originating from PDAC cells (Fig. 6E).

## Discussion––Enhance the infiltration capacity of CAR-T cells by emulating the strategy of macrophage infiltrating the tumor microenvironment

**Fig. 7:**
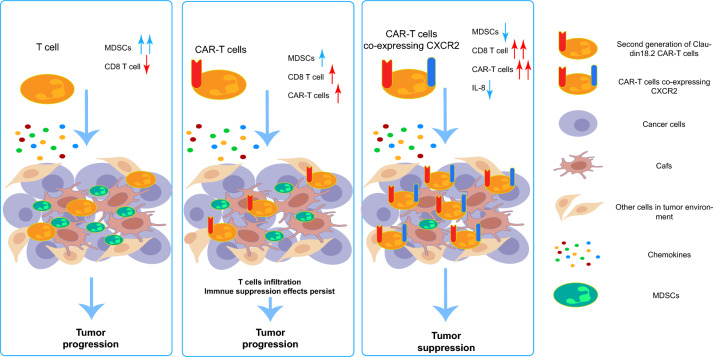
Graphic abstract. The left figure: In patients with pancreatic cancer, T cells demonstrate a capacity for infiltrating the pancreatic cancer microenvironment. However, this ability is hindered by the fibrotic nature of pancreatic cancer. Concurrently, MDSCs inhibit these infiltrating T cells, diminishing their tumor-destroying capabilities and ultimately facilitating tumor progression. The central figure: After administering CAR-T cells to patients with pancreatic cancer, these CAR-T cells possess a superior ability to infiltrate and recognize tumor cells compared to standard T cells. Nonetheless, the immunosuppressive network established by MDSCs alongside other suppressive cells within the immune microenvironment of pancreatic cancer remains unaltered. As a result, CAR-T cells are prevented from executing their normal tumor-killing function. The right figure: The introduction of CXCR2 into CAR-T cells led to the creation of CXCR2 CAR-Ts that inhibited the migration of MDSCs and CXCR2 macrophages to the pancreatic cancer site. This resulted in lower levels of IL-8 in the microenvironment, enabling CAR-T cells to enjoy enhanced infiltration abilities. This process also improved the composition of cells and factors within the pancreatic cancer microenvironment, thereby alleviating, to a certain degree, the immunosuppressive environment of pancreatic cancer. This ultimately resulted in the inhibition of in situ tumor formation and metastasis

Despite promising results from preclinical studies, numerous obstacles persist in the use of CAR-T therapy for solid tumors. In previous research, we found that neither tumor-infiltrating lymphocytes (TILs) treatments nor CAR-T therapies were able to halt tumor progression (Fig. 7, left and middle). To enhance CAR-T cell infiltration into PDAC tissues, we designed and screened for CXCR2 CAR-T targets. We demonstrated that CXCR2 CAR-T could modify the constitution of immunosuppressive cells within the PDAC immune microenvironment. Moreover, we observed changes in the levels of immunosuppressive cytokines, such as IL-8 (Fig. 7, right). In summary, CXCR2 CAR-T cell therapy significantly improved CAR-T cell infiltration into tumor tissues and reshaped the cellular composition within the PDAC immune microenvironment, thereby enhancing antitumor effects.

Chemokines are a group of cytokines with broad functions in controlling cell migration. The infiltration degree of T cells and other types of immunosuppressive cells into tumor tissues largely depends on the compatibility between the chemokines secreted by tumor tissue components and the chemokine receptors on immune cells. CXCL5, a major ligand of CXCR2, was previously shown to be associated with the degree of infiltration of immune cells in PDAC [23, 24]. Studies have also validated the effectiveness of CXCR2 inhibitors as an antitumor strategy [25, 26]. In this study, we examined the expression of the chemokine family in PDAC tissues and explored the spatial relationship between CXCR2 and its ligands in these tissues. We found that a subpopulation of CXCR2-positive macrophages was specially expressed in PDAC, which could potentially develop into a novel therapeutic strategy. Furthermore, flow cytometry confirmed the lack of CXCR2 expression in peripheral circulating blood and TILs in patients with PDAC. Given the fact that macrophages occupy a significant portion of the immune microenvironment in PDAC, we believe that understanding the infiltration strategies of macrophages may enrich CAR-T treatment strategies for PDAC infiltration. In addition to the influence of chemotactic factors, macrophage infiltration in pancreatic cancer is also regulated by TLS and inherent pancreatic tissues such as adipose tissue. As such, we engineered CXCR2 CAR-T therapy, which not only enhanced infiltration under the unique environment of PDAC stroma but also reshaped the cellular composition in the PDAC immune microenvironment to some extent. This hindered the infiltration of MDSCs and CXCR2 + macrophages, alleviated immunosuppression in the PDAC microenvironment, and ultimately enhanced the antitumor effects of CAR-T. Previous studies have found that chemokine expression is highly correlated with PDAC metastasis [27, 28]. As PDAC is a highly metastatic cancer type, it is important to verify the effects of CAR-T in treating metastatic tumors besides limiting the growth of primary tumors. The unexpected therapeutic effects of CXCR2 CAR-T in the PDAC liver metastasis model may be due to the more exposed recognition of CXCL5 in metastatic tumors compared to the shielding of the stroma structure in primary tumors. This allows CAR-T to better locate tumor positions. The recruitment of immunosuppressive cells in metastatic tumors is also less than in primary tumors. However, the specific molecular mechanism still needs to be explored.

Based on the findings, we propose a therapeutic strategy for PDAC CAR-T research. By studying the recruitment strategies of macrophages, important constituents of the PDAC immune microenvironment, and giving CAR-T cells similar migration patterns, we can enhance the infiltration and retention ability of CAR-T cells, thereby achieving better antitumor effects. Previous studies of PDAC CAR-T mainly focused on CXCR4 and CXCR6. CXCR4 has also been proven to be one of the important ways for macrophages to infiltrate PDAC [19, 29, 30]. The superior efficacy of CXCR4 CAR-T over CXCR6 may be attributed to the unintentional alignment of the CAR-T cell infiltration strategy with the macrophage infiltration pattern.

In conclusion, our study provides evidence that co-expression of CXCR2 in CAR-T cells can enhance antitumor effects, increase T-cell recruitment and proliferation at PDAC tumor sites, and does not produce cellular toxicity. Our findings may offer a potential therapeutic strategy for the treatment of PDAC.

## Materials and methods

### Human specimens

Tumor and blood samples were obtained from patients who underwent surgical resection of PDAC at Fudan University Shanghai Cancer Center. All specimens were confirmed by postoperative pathology. The experiments were conducted under protocols approved by the Ethics committee of Fudan University Shanghai Cancer Center, and written informed consents from all patients and healthy donors were obtained.

### Mice

Male C57BL/6 mice, aged eight weeks, were maintained in the Specific Pathogen Free (SPF) facility at the Cancer Institute Technology Platform. For the subcutaneous tumor model, 1 × 106 KPC or PANC02 cells were suspended in 100 μl of PBS (pH 7.4). Approximately 14 days later, once the average tumor size attained 60 mm3, mice with excessively large or small tumors were excluded. Mice with comparable tumor sizes were then randomly assigned to groups for cell treatment. A total of 2 × 106 CAR-T cells were administered via tail vein injection. We monitored the mice's body weight and tumor measurements, calculating tumor volume (mm3) using the formula (length × width^2^)/2. We observed the mice for signs of cytokine release syndrome, and euthanized them on day 28. Tumors were collected and partially dissociated into single-cell suspensions for flow cytometry analysis, while the remaining portions were fixed in formaldehyde for immunohistochemistry. In the liver metastasis model, 1 × 106 KPC cells were administered through splenic surgery. Cell treatment was also undertaken on day 14. Based on survival experiments, day 28 was identified as a critical time point in the metastatic tumor model, exhibiting significant divergence in survival. This time point was subsequently used for histochemical analysis and comparison. All animal experiments were conducted according to the protocol approved by the Ethics Committee of the Shanghai Cancer Prevention and Control Center at Fudan University.

### Cell lines culture, cell purification and differentiation in vitro

PDAC cell lines used in this study included PANC02 and KPC. All of the cell lines used in this study were purchased from ATCC and were authenticated by The Cell Bank of Type Culture Collection of the Chinese Academy of Science (CBTCCCAS) with STR profiling. Cells were cultured with DMEM or 1640 medium (Gibco, China) with 10% fetal bovine serum and 1% penicillin/streptomycin in 37 °C incubator and 5% CO_2_ culture conditions.

#### Vector constructs, lentivirus production and in vitro transduction

The sequences for the single-chain variable fragment (scFv) were inserted upstream of the hinge and transmembrane structural domains. This was followed by the inclusion of the 4-1BB co-stimulatory structural domain and the CD3ζ signaling structural domain. For the CXCR2 CAR-T, mouse CXCR2 (mCXCR2) was also linked via the F2A peptide sequence. These constructs were then subcloned into a lentiviral vector backbone. Lentiviral vectors were prepared by co-transfecting the constructs with packaging plasmids PAX2 and pMD2.G into HEK-293 T cells using Lipofectamine 3000. After 48 and 72 h, the cell culture supernatants, which contained the lentiviral particles, were harvested and subsequently concentrated using Amicon Ultra-15 centrifugal filters (Millipore, China). The overexpression efficiency of either CXCR2 or the CAR constructs was assessed 48 h post-infection.

#### Transwell

In the experimental group, cells (5 × 10^5^) were seeded into Transwell chambers (3 μm, Costar, Corning Inc., Corning, NY, USA). The tumor culture supernatant, which had been previously filtered to remove any cellular debris, was subsequently added to the bottom of these chambers. The migration of cells through the chambers was then evaluated after incubation periods of 2 h and 6 h.

#### Flow cytometry and antibodies

In general, cell surface staining was performed in ice-cold PBS containing 0.5% BSA for 30 min at 4 °C. Non-specific staining was blocked using Fc receptor blocker (BD Biosciences). Dead cells were excluded using the fixable viability dye eFluor780 (eBioscience, USA). Transcription factor staining was performed after using the Foxp3 Fixation and Permeabilization Kit (eBioscience). Surface molecule staining was performed before fixation. For intracellular cytokine staining, cells were stimulated in vitro for 6 h with Leukocyte Activation Cocktail (BD Biosciences) and stained according to the manufacturer's instructions. The following antibodies were used: live/dead (FVS780), anti-CD45 (HI30, 13/2.3), anti-CD4(RM4.5), anti-CXCR2 (SA0446), anti-CD3*ε* (152,303), anti-CD8*α* (53–6.7), anti-CD11c (B-ly6), anti-CD86 (CL-1), anti-G4S linker (GS-ARAP25), anti-Gr-1 (RB6-8C), anti-CD206 (C068C2), anti-CD62L (MEIL-14), anti-CD44 (IM-7)and isotype controls (MOPC-21,27–35,X40,R35-95). All antibodies were purchased from BD Bioscience and Biolegend (San Diego, USA).

#### Cytotoxicity Assay

The cytotoxicity of CAR-T cells was assessed using a lactate dehydrogenase (LDH) assay kit (Solarbio, China). Initially, 1 × 10^4^ PDAC cells (either KPC or PANC02) were introduced into the lower compartment of a Transwell plate. After a 12 h period, which ensured adhesion of PDAC cells to the culture dish, the T cells, CAR-T cells, or CXCR2 CAR-T cells were added either directly to the co-culture system or to the upper chamber, subsequent to the uniform distribution of matrix gel at its bottom. Upon completion of 48 h, the cells were harvested for subsequent analyses. Cytotoxicity was computed using the following formula: Cytotoxicity (%) = [(Mixed cell assay—effector cell spontaneous—target cell spontaneous—medium control)/(target cell maximum—target cell spontaneous—medium control)] × 100%.

#### ELISA

Mouse sera were collected on day 28(14 days after treatment with CAR-T cells). IL-2,IL-4, TNF*α*, IFN-*γ* and IL-8 in serum were quantified according to the manufacturer’s directions of the ELISA kits.

#### Statistical analysis

Comparisons between two different groups were performed using unpaired two-tail Student’s t test. Linear regression methods were applied for correlations when appropriate. *P* values were calculated using correlation coefficients and sample sizes. Overall survival of tumor bearing mice was analyzed using the Kaplan–Meier method log-rank test. Data were presented as means ± SDs. Two-sided P values less than 0.05 were considered statistically significant, and the asterisks indicated the significance level of the *p* value: *p < 0.05, **p < 0.01 and ***p < 0.001). Statistical analyses were performed with Prism software (version 6, GraphPad Software, Inc.

### Fluorescent mIHC

Fluorescent mIHC was performed in serial sections of FFPE tumor tissue from each PDAC patient using the Opal 7-Colour Manual IHC Kit (PerkinElmer Hopkinton, Massachusetts, USA) according to the manufacturer’s protocol and as described in our previous article. In short, sections experienced primary antibodies including Rabbit monoclonal antibodies anti-CD68 (1:500, Abcam, Cambridge, MA, USA), anti-CXCR2 (1:500, Abcam), anti-CD206 (1000, Abcam), anti-CXCL1 (1:200, Abcam), anti-CXCL5 (1:200, Abcam), anti-CXCL8 (1:500, Abcam) and anti-CD8 (1:2000, Abcam), followed by HRP-conjugated secondary antibody and fluorescent dyes (Opal520, Opal570, Opal620, Opal690, and DAPI). Stained sections were scanned and processed by a Vectra PolarisTM Automated Quantitative Pathology Imaging System (AKOYA Biosciences, PerkinElmer, Massachusetts, USA).

#### Organoid preparation and acridine orange/propidium Iodide (AO/PI) staining

Surgical-resected PDAC specimens for organoid preparation were immersed in cold PBS saline (Basal Medium # B310KJ) with 1% Penicillin/Streptomycin (Basal medium # S110JV). After cutting and digestion via the Solo Digestive Enzyme Kit (Sinotech Genomics # JZ-SC-58201) for 1–2 h, the cell mixture was filtered through a 70 μm sieve and centrifuged at 300 g for 5 min. The pellet was embedded in GFR Matrigel (R&D systems #3533–005-02) and cultured in a complete organoid expansion medium containing RSPO1 (Peprotech #120–38), WNT3A (Peprotech #315–20), NOGGIN (Peprotech #120-10C), FGF10 (Peprotech #100–26), EGF (Peprotech # AF-100–15) and other supplements as previously described. Organoids were generated and passaged for 3–5 generations and then cryopreserved. The recovered organoids used in our study are between the 7th and 10th generations, and the culture medium is changed every 4 days. For co-culture of organoids and T cells, tumor organoids were partially digested from Matrigel to retain the three-dimensional architecture. The remaining Matrigel was washed before coculture. Cell count and viability were performed via ViaStainTM AO/PI staining (Nexcelom #CS2-0106) on living organoids. The stained organoids were imaged under Confocal laser scanning microscopy (Olympus FV3000) and quantified via Fiji (Image J) software.

### Supplementary Information

Below is the link to the electronic supplementary material.

Supplementary file1 (PDF 8256 KB)

## Data Availability

The results of the in vitro functional experiments were replicated three times, and the results of the animal experiments were not subjected to any subjective outcome selection.
