# Supplementary figures and images for: Ectopic CXCR2 expression cells improve the anti-tumor efficiency of CAR-T cells and remodel the immune microenvironment of pancreatic ductal adenocarcinoma
Source: Cancer Immunol Immunother. 2024 Mar 2;73(4):61. doi: 10.1007/s00262-024-03648-y (PMC10908625; doi:10.1007/s00262-024-03648-y)

**A**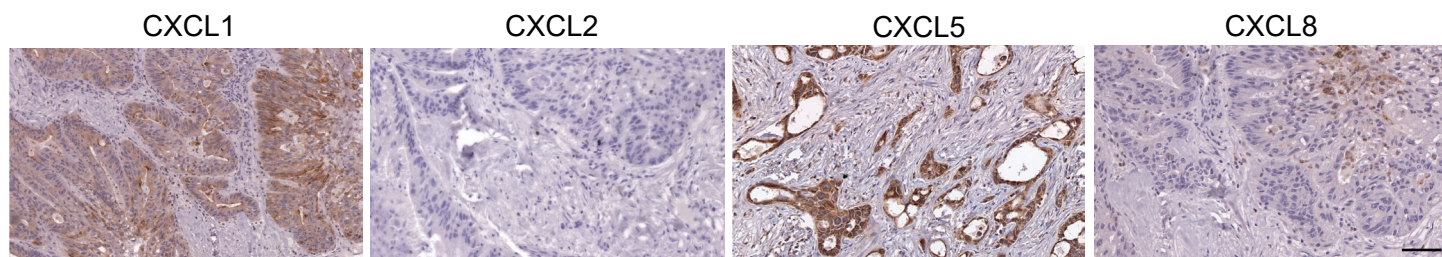**B**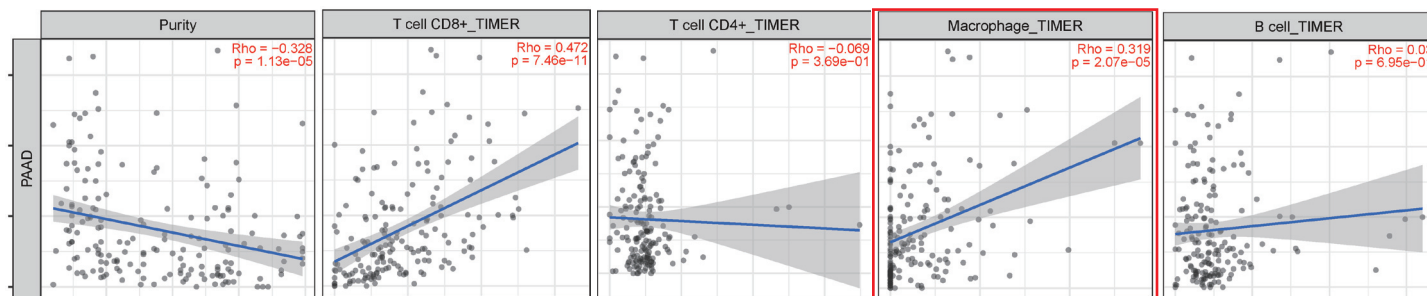**C**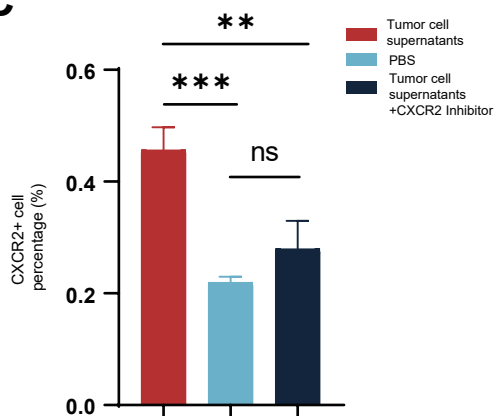**D**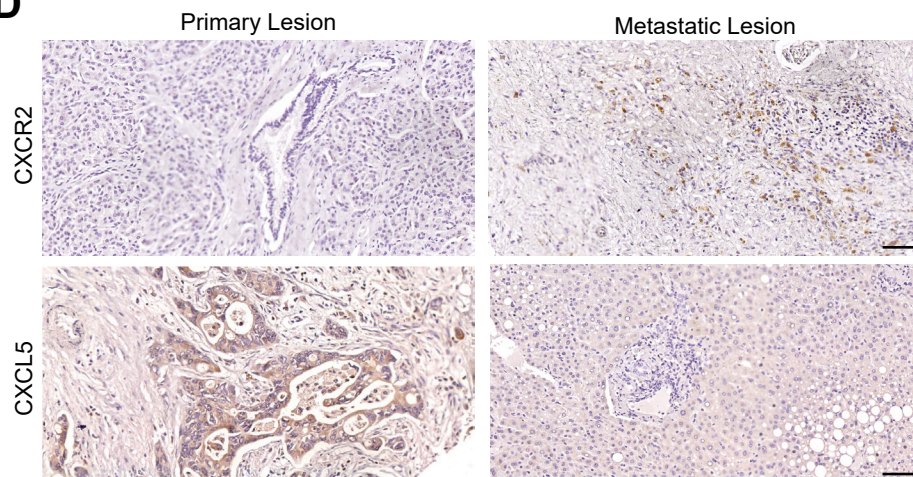**E****PANC02 tumor**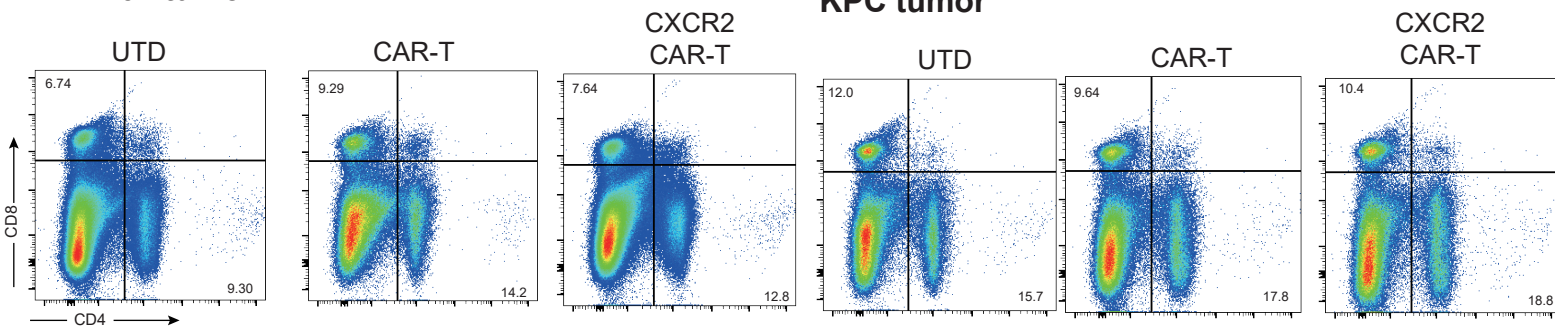**KPC tumor**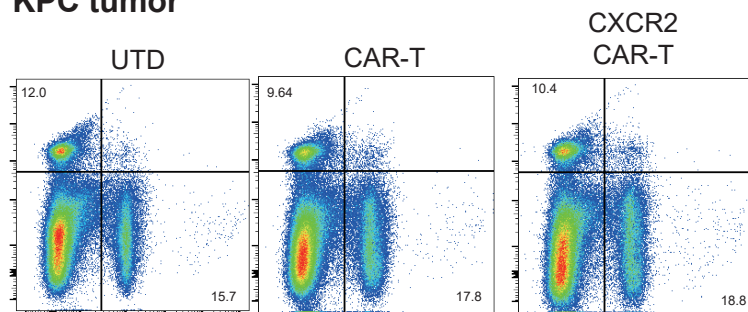

Supplement: Supplementary file 1 — Supplementary file1 (PDF 8256 KB) [file 262_2024_3648_MOESM1_ESM.pdf]
